# Diversity and seasonality of host-seeking ticks in a periurban environment in the Central Midwest (USA)

**DOI:** 10.1371/journal.pone.0250272

**Published:** 2021-04-23

**Authors:** Ali Hroobi, Gunavanthi D. Boorgula, David Gordon, Jianfa Bai, Doug Goodin, Gary Anderson, Savannah Wilson, Alex Staggs, Ram K. Raghavan

**Affiliations:** 1 Department of Biology, College of Science, Al-Baha University, Al-Baha, Kingdom of Saudi Arabia; 2 Department of Diagnostic Medicine and Pathobiology, College of Veterinary Medicine, Kansas State University, Manhattan, Kansas, United States of America; 3 Department of Biology, Pittsburg State University, Pittsburg, Kansas, United States of America; 4 Department of Geography, College of Arts and Sciences, Kansas State University, Manhattan, Kansas, United States of America; 5 Medgene Labs, Paola, Kansas, United States of America; 6 Center for Vector-Borne and Emerging Infectious Diseases, Department of Veterinary Pathobiology, College of Veterinary Medicine, University of Missouri, Columbia, Missouri, United States of America; 7 Department of Public Health, School of Health Professions, University of Missouri, Columbia, Missouri, United States of America; Tufts University Cummings School of Veterinary Medicine, UNITED STATES

## Abstract

Between March 2014 and February 2017, host-seeking ticks were collected during the late spring and summer months seasonally, and as well as continually through all seasons from several sites in a periurban environment in Pittsburg, Kansas, located in the Central Midwestern United States. All three post-emergent life-stages of *Amblyomma americanum*, and the adults of three other ticks viz. *Dermacentor variabilis*, *A*. *maculatum*, and *Ixodes scapularis* were collected using the flagging method, and were taxonomically identified using morphological and molecular methods. A total of 15946 ticks were collected from these sites. A vast majority of the ticks collected over the three-year study period was *A*. *americanum* (79.01%). The three other species collected included *D*. *variabilis* (13.10%), *A*. *maculatum* (7.15%), and *Ixodes scapularis* (0.73%). More female ticks of each species were collected throughout the study period from all sites, and a unimodal activity period was noted for all four species. The diversity, composition, and phenology of these medically significant tick species are discussed.

## 1. Introduction

Tick-borne diseases (TBDs) to humans, livestock and domestic pets in the central Midwestern US are increasing in their frequency and intensity; recent studies using retrospective public health department data and historical data gathered at veterinary diagnostic facilities indicate that the spatial and spatiotemporal prevalence of cases of feline tularemia [1], cytauxzoonosis [2], human monocytic ehrlichiosis [3], Rocky Mountain spotted fever (RMSF) [4], and bovine anaplasmosis [5] have worsened over the past several years, at least partially owing to various environmental and climate related factors. Studies have also shown that the suitability for the spatial distribution of ticks in this region are broader than previously thought [6–10], whose distributions are also influenced by climatic and environmental factors. An explosion of white-tailed deer (*Odocoileus virginianus*) population in N. America, the primary vector for *Amblyomma americanum* ticks in the region [11] during the recent decades is also an added factor contributing to the present TBD dynamics.

The southeastern parts of Kansas in particular but generally those counties neighboring the states of Missouri, Oklahoma and Arkansas regularly report higher than average number of TBD cases to the state health departments [12]. Retrospective evaluations of case and diagnostic data from this region have identified an area covering several contiguous counties as a hot-spots for TBDs [3, 5]. The prevention and management of TBDs rely upon the fundamental knowledge of what ticks are present in a given area and the times in which they are likely to come in contact with their accidental hosts.

Earlier studies provide some guidance on the seasonality and types of tick species that may be expected in the broader study region (Kansas, Missouri, Oklahoma, Arkansas) (Table 1). The list provided in Table 1 is not exhaustive, and ticks in these studies have been collected from different sources; including those ticks attached to different wildlife and domestic hosts, and free-living ticks that were collected by dragging, flagging and CO_2_ traps. The risk of contracting TBDs by humans and pet animals is higher from contact with host-seeking (or) questing ticks. However, the diversity, activity period beyond the tick season, or the overlaps in emergence periods for different life-stages of host-seeking ticks in the region are only rarely available. A recent study in Oklahoma by Small et al. [13], continuously collected ticks from two habitats each month between July 2016 through July 2017 wherein the seasonality of three tick species is reported. Rynkiewicz and Clay [14] documented tick species composition and seasonality in several sites in the state of Indiana, also in the Midwestern region. However, there are notable differences in the climate and geography in these study sites, and tick life history characteristics can change with changes in climate and habitats.

**Table 1:** Previous tick phenological studies in the region.

Study	Tick source	Ticks identified	Region
Tugwell and Lancaster (1963) [15]	Wildlife hosts	*Amblyomma americanum*, *Ixodes scapularis*, *I*. *cookei*, *Haemaphysalis leporispalustris*, *Dermacentor variabilis*, and *I*. *dentatus*	NW Arkansas
Semtner and Hair, 1973 [16]	Free-living and host-attached ticks	*A*. *maculatum*	Northeastern and central Oklahoma
Koch (1982) [17]	Domestic dogs	*A*. *americanum*, *Rhipicepphalus sanguineus*, *D*. *variabilis*, and *I*. *scapularis*	Northwestern Arkansas and southeastern Oklahoma
Brillhart et al. (1994) [18]	Small and medium-sized mammals	*A*. *americanum*, *D*. *variabilis*, *H*. *leporispalustris*, *I*. *cookei*, *I*. *kingi*, *I*. *sculptus*, and *I*. *texanus*	Kansas
Kollars et al (2000a) [19]	Free-living, vertebrate hosts	*D*. *variabilis*	Missouri
Kollars et al. (2000b) [20]	Free-living, vertebrate hosts	*A*. *americanum*	Missouri
Mock et al., (2001) [21]	Wild turkey	*A*. *americanum*	Eastern one-third of Kansas
Kollars and Oliver (2003) [22]	Free-living, vertebrate hosts	*H*. *leporispalustris*, *I*.*brunneus*, *I*. *cookei*, *I*. *dentatus*, and *I*. *texanus*.	Missouri
Barker et al. (2003) [23]	Domestic cattle, wildlife	*A*. *maculatum*	Oklahoma
Brown et al. (2011) [24]	Free-living	*A*. *americanum*, *D*. *variabilis*, *A*. *maculatum*, *I*. *brunneus*	Missouri
Bouzek et al. (2013) [25]	Free-living	*A*. *americanum*	Northeast Missouri
Kaizer et al. (2015) [26]	Free-living	*A*. *americanum*	Northeast Missouri
Mangan et al. (2018) [27]	Free-living	*A*. *americanum*	Northeast Missouri
Small et al. (2019) [13]	Free-living	*A*. *americanum*, *D*. *variabilis*, *A*. *maculatum*, *I*. *scapularis*	Oklahoma
Hahn et al. (2019) [28]	Host attached, free-living	*I*. *scapularis*	Northeast Missouri

The objective of this study was to evaluate the current diversity and seasonality of host-seeking ticks from an area in SE Kansas surrounding a periurban environment frequented by humans and their pets, and as well from areas classified as forested and grassland landscape. Information gathered in this study will assist in public health planning in the region, and it will also serve as a baseline for comparing future tick diversity and population dynamics influenced by non-stationary, anthropogenic factors such as climate change.

## 2. Materials and methods

Permission to collect ticks for this study were obtained from private landowners. The Kansas Department of Wildlife, Parks and Tourism was notified of this study, which determined that permission to conduct the study was not required. No endangered or protected species were involved in this study.

### 2.1 Study area

**Fig 1:**
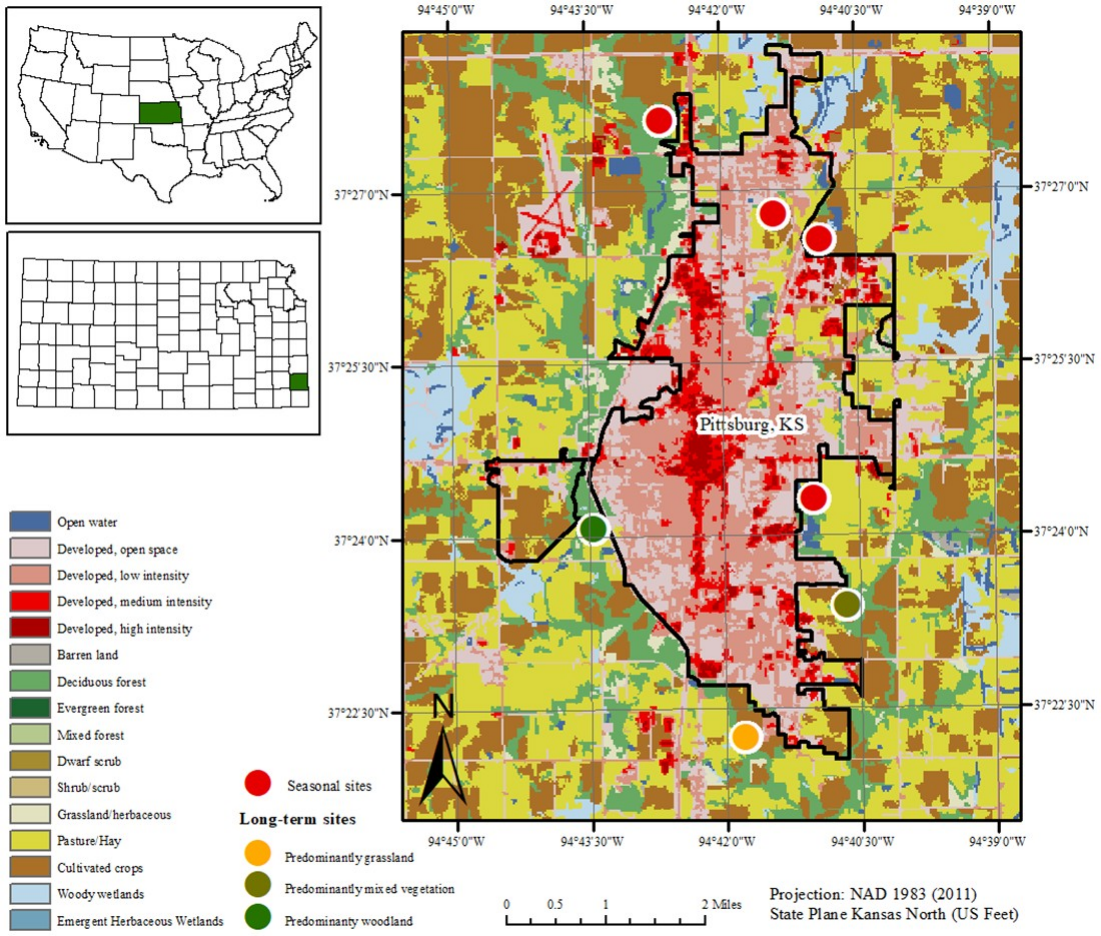
Short and long-term tick collection sites around Pittsburg, USA.

This study was conducted in south-eastern Kansas, around periurban areas surrounding the city of Pittsburg (37.4109° N, 94.7050° W) (Fig 1). Pittsburg is located in Crawford county with an estimated population of 20,178 people living in 2018 (US Census, 2020). Pittsburg and the area around it sit in the Ozarks highland ecoregion characterized with a mix of prairie and forest cover. Climate in Pittsburg is humid and subtropical (Köppen Climate Classification), summers are hot and humid with temperatures with as many as 73 mornings staying above 20° C. Falls are cooler with temperatures ranging between 10–15°C, winters are highly variable ranging from -17° C to 21°C depending on artic outbreaks and moist air from the Gulf. Temperatures can vary erratically during spring with temperatures generally rising in late March. Much of the rainfall in this area occurs during the spring, with up to 514.1 mm of rain received between March and June. It also rains during the fall, with up to 222.8 mm recorded. Winter weather in Pittsburg is less dry relative to other parts of Kansas owing to moist air that moves in from the Gulf of Mexico.

### 2.2 Tick collection and identification

There were two primary objectives in the study; the first was to determine the tick diversity in the study region, which was evaluated by collecting host-seeking ticks from four locations between the months of March and August. Ticks were collected following the flagging method [29], using a 1 m^2^ white flannel cloth attached to a 1.3 m long flagpole. Ticks attached to the flannel were picked using forceps at every 2–5 m distance and stored in a plastic container, placed on dry ice until they were brought to the laboratory, where they were placed in -20° C for 48–56 hours. Following this period, they were thawed on countertop and microscopically identified to their species level using N. American tick taxonomic keys [30]. Tick surveys were performed by four similarly trained biologists during the entire study period, but tick collectors changed once in the second year of the study. Ticks were collected from three approximately 100 m long linear transects that were separated by varying distances (50–100m) in different study sites. Nymphs and larvae were first identified using morphological keys, and to further confirm the species, a subset of these ticks was evaluated using molecular analysis.

Secondly, to study the phenology of ticks, three study sites were selected, each representing a dominant land cover type; predominantly woodland, predominantly grassland, and mixed vegetation area, to represent variation in tick densities in different land cover types. From these sites, ticks were collected from 300 m^2^ transects, once a month from the beginning of March 2014 through the end of March 2017, as described above, except for periods when the transects were under snow cover and/or temperature was below freezing. No time limit was set and entire transects were swept for ticks over the vegetation cover. Once collected, ticks were handled as described above. Ticks were identified morphologically and cross-checked with molecular methods. This data is available upon request.

## 3. Results

**Fig 2:**
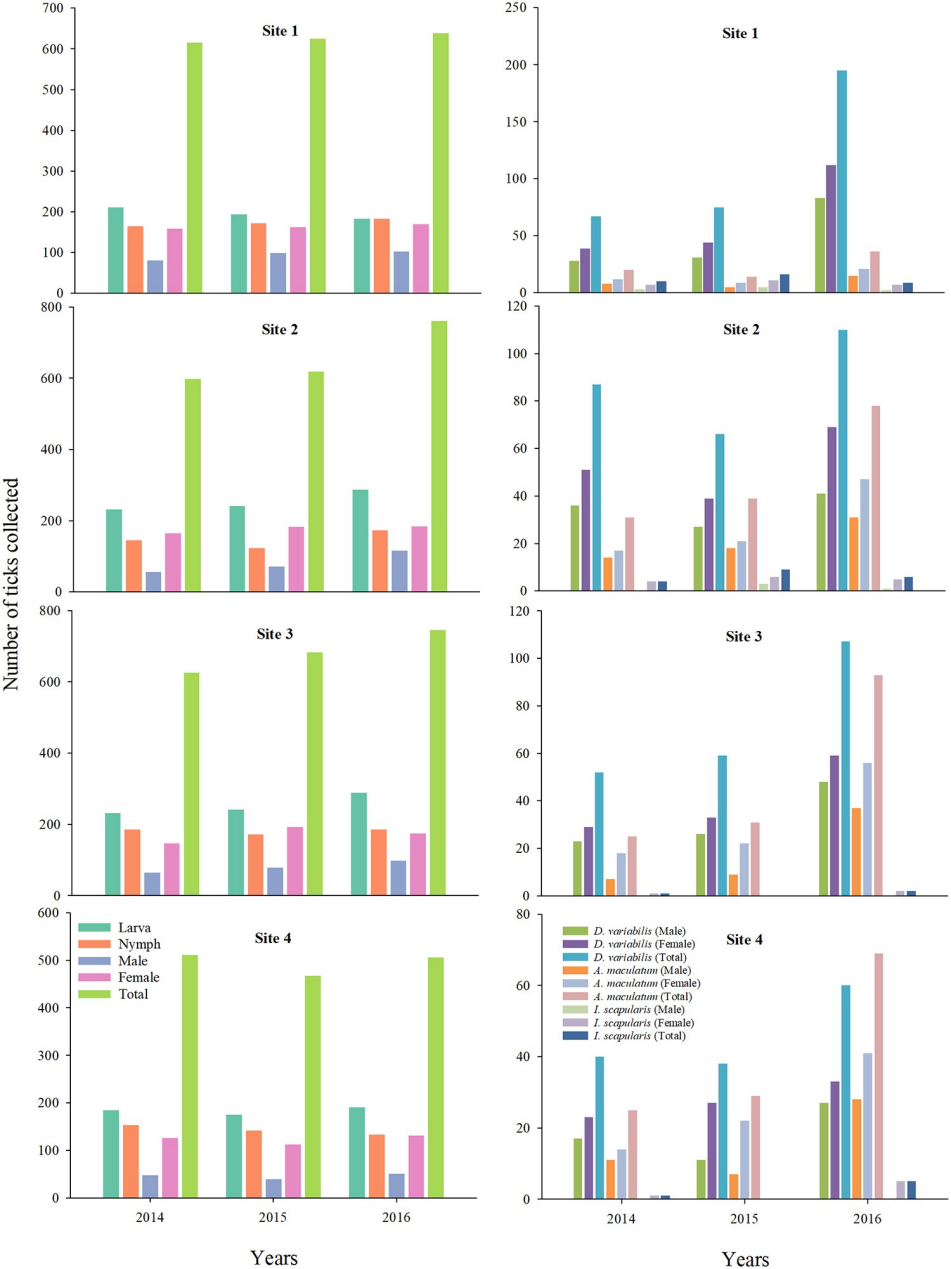
Tick diversity and composition observed in four seasonal sites around Pittsburg, Kansas between years 2014–2017. Plots in the left panel depict the number of three post-emergent stages and sexes of *Amblyomma americanum* collected, and plots in the right panel depict the number of male and female sexes of *D*. *variabilis*, *A*. *maculatum*, and *I*. *scapularis* collected.

Between March 2014 and February 2017, a total of 15946 ticks were collected from sites around the city of Pittsburg, Kansas. Of these, 7043 ticks were collected from three long-term sites, and 8903 ticks were from four seasonal sites. A vast majority of the ticks collected from the seasonal sites over the three-year study period was *A*. *americanum* (n = 7394; 83.05%). The three other species collected from these sites included *D*. *variabilis* (n = 956; 10.73%), *A*. *maculatum* (n = 490; 5.50%), and *Ixodes scapularis* (n = 36; 0.40%). All four questing life-stages of *A*. *americanum* ticks were collected; larva (n = 2657; 29.84%), nymph (n = 1931; 21.68%), and adult (male: n = 904; 10.05%; female: n = 1902; 21.36%). There were 956 adult *D*. *variabilis* ticks collected by flagging, which included 398 males (4.47%) and 558 females (6.26%). Among the 490 *A*. *maculatum* ticks, there were 190 males (2.13%), and 300 female ticks (3.36%). Questing adults of *I*. *scapularis* ticks were collected from all sites in the three years (male: n = 14; 0.15%; female: n = 49; 0.55%). Although there were differences in the total numbers, all four species of ticks were collected from all study sites. The number of ticks collected, by species, year and individual seasonal sites is present in (Fig 2) (S1 File). In all sites and in all years, more female ticks of the four species were collected than their male counterparts; and, there were inter-annual variations in the male:female ratio for the different species in the seasonal sites (Table 2).

**Table 2:** Male:Female ratios of tick species collected seasonally from sites located near Pittsburg, Kansas between years 2014–2016.

Species	2014	2015	2016	Overall
*Amblyomma americanum*	0.41:1	0.44:1	0.55:1	0.47:1
*Dermacentor variabilis*	0.73:1	0.66:1	0.72:1	0.71:1
*Amblyomma maculatum*	0.65:1	0.52:1	0.67:1	0.63:1
*Ixodes scapularis*	0.23:1	0.47:1	0.15:1	0.28:1

**Fig 3:**
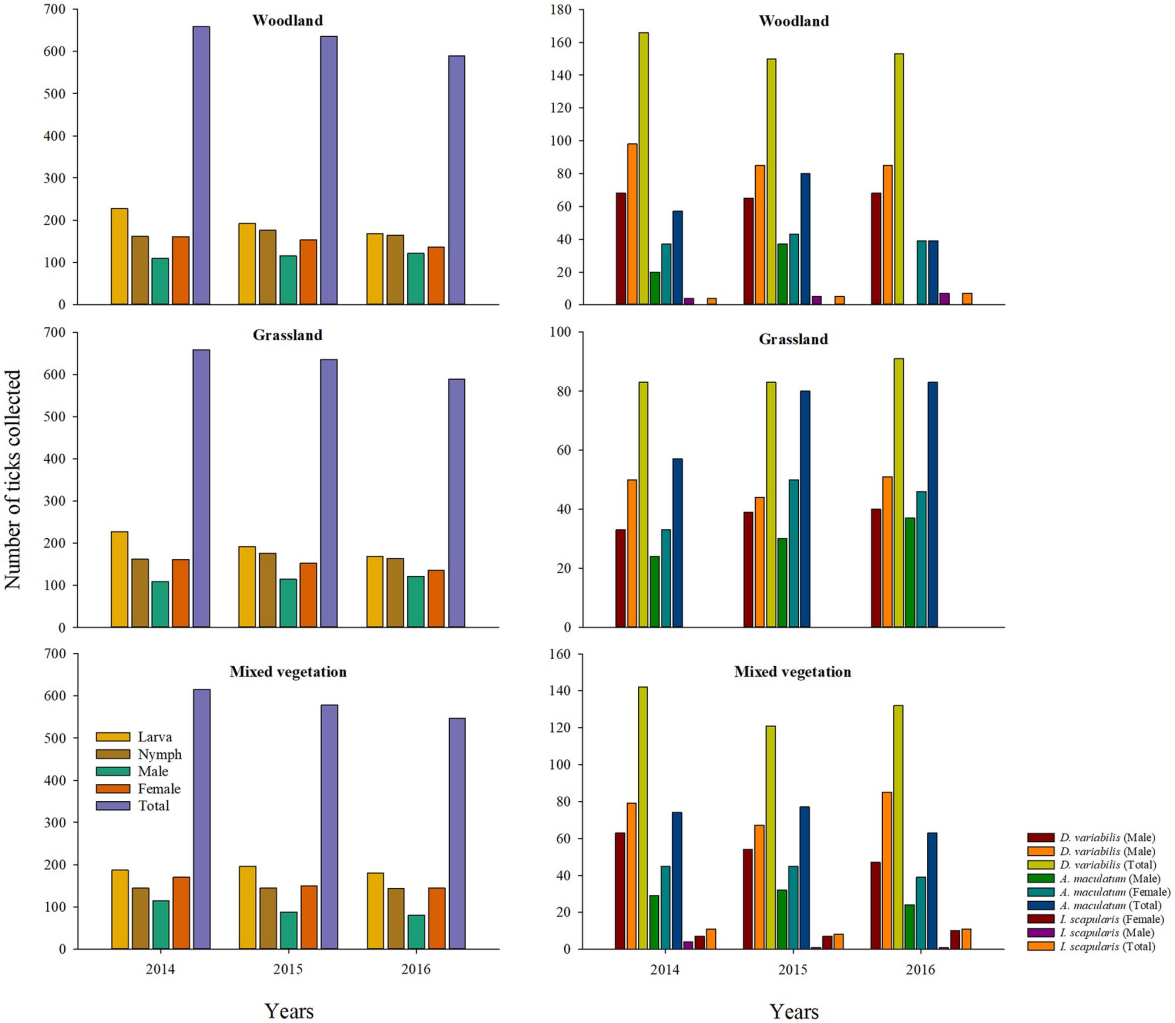
Tick diversity and composition observed in three long-term sites around Pittsburg, Kansas between years 2014–2017. Plots in the left panel depict the number of three post-emergent stages and sexes of *Amblyomma americanum* collected, and the plots in the right panel depict the number of male and female sexes of *D*. *variabilis*, *A*. *maculatum*, and *I*. *scapularis* collected.

Relatively more females of *D*. *variabilis* and *A*. *maculatum* were collected compared to *A*. *americanum*, and far fewer males of *I*. *scapularis* were present in these sites, although this ratio was based only on 63 ticks. The diversity and composition of questing tick species from the three long-term sites were similar to those of the seasonal sites (Fig 3).

**Fig 4:**
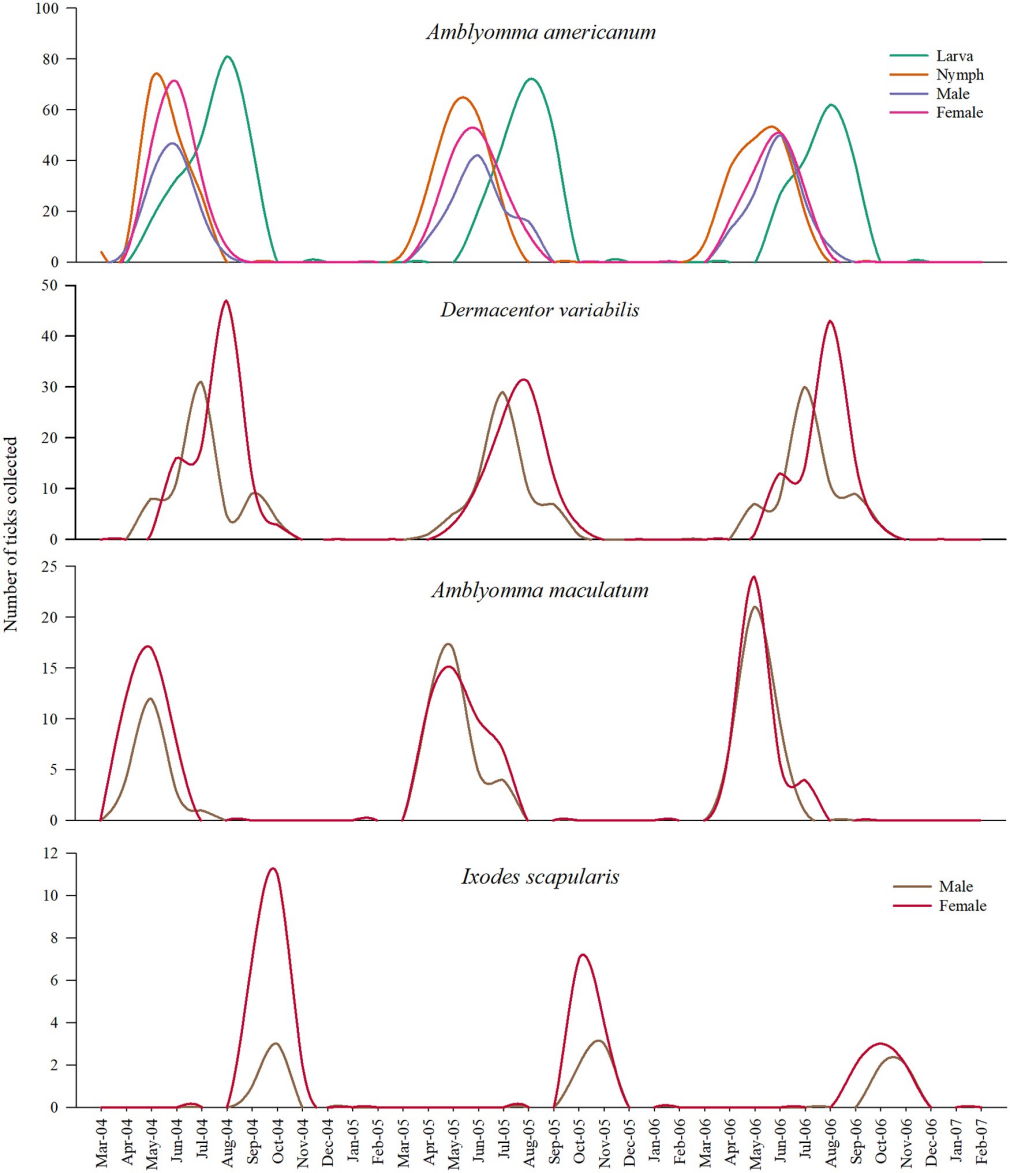
Tick phenology observed in predominantly woodland vegetation near Pittsburg, Kansas between years 2014–2017. Lines represent total number of individual specimens collected at monthly intervals and are not smoothed estimates.

**Fig 5:**
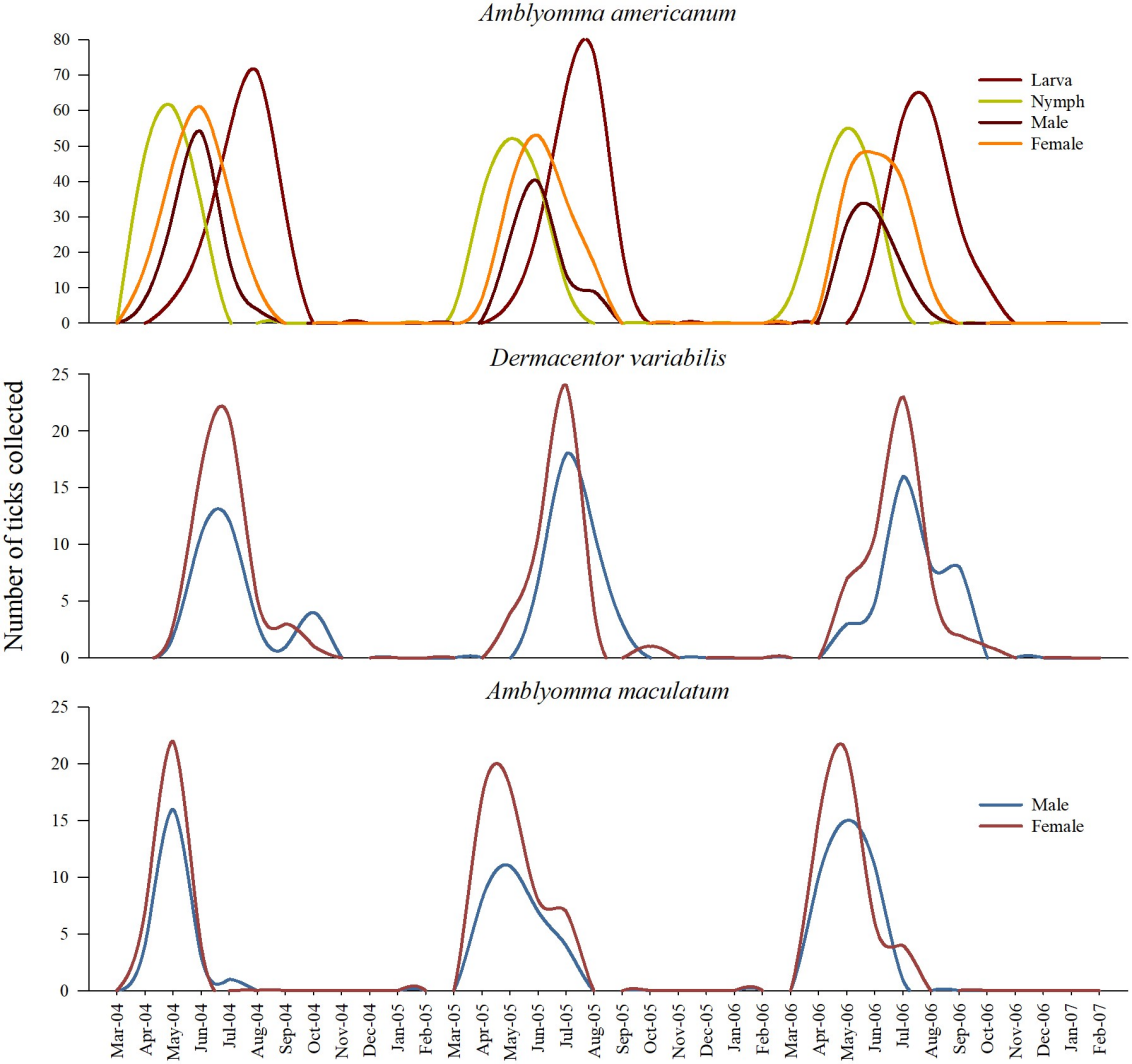
Tick phenology observed in predominantly grassland vegetation near Pittsburg, Kansas between years 2014–2017. Lines represent total number of individual specimens collected at monthly intervals and are not smoothed estimates.

**Fig 6:**
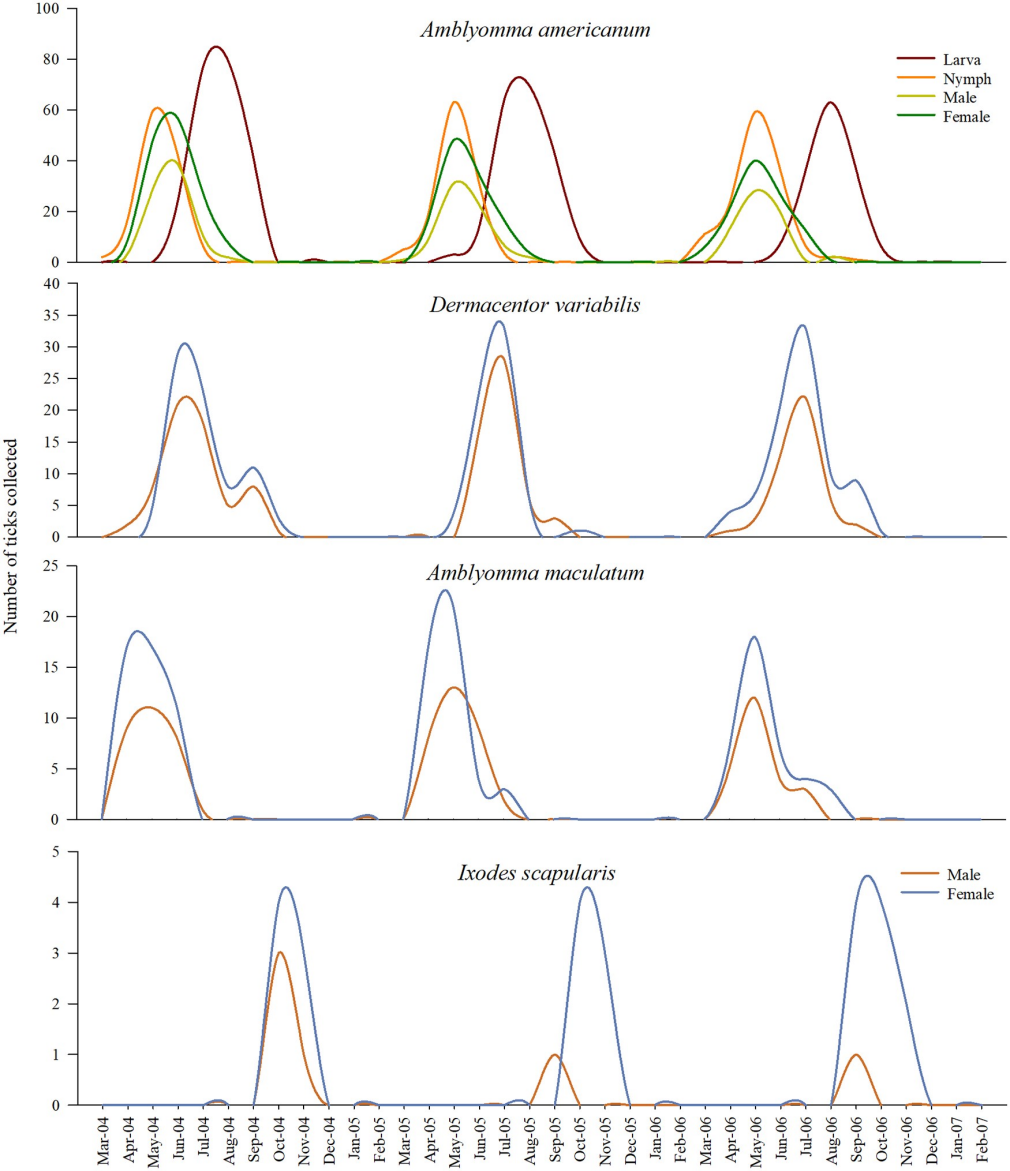
Tick phenology observed in mixed vegetation near Pittsburg, Kansas between years 2014–2017. Lines represent total number of individual specimens collected at monthly intervals and are not smoothed estimates.

A total of 7043 individual ticks were collected from the long-term sites, which included, *A*. *americanum* (male n = 847, female n = 1291, nymph n = 1322, larva n = 1725); and the adults *of D*. *variabilis* (n = 1126; male = 477, female = 649), *A*. *maculatum* (n = 651; male = 272, female = 379), and *I*. *scapularis* (n = 81; male = 19, female = 62) ticks. The number of ticks collected, by species, year and the individual long-term study sites is present in Figs 4–6, S2 File. Similar to the seasonal sites, all species of ticks were collected from the three long-term sites. There were more females than males for all species in all sites throughout the study period. There were more male *A*. *americanum* ticks found in the long-term sites relative to the seasonal sites. The overall (all sites, all years combined) male:female ratio of *A*. *americanum* ticks were 0.65:1 and the ratios were 0.63:1, 0.65:1, and 0.68 for years 1–3, respectively. Relatively more females of *D*. *variabilis* were collected with an overall ratio of 0.73:1 (year 1 = 0.72:1; year 2 = 0.80:1; year 3 = 0.68:1). The overall male:female ratio for *A*. *maculatum* was 0.71:1 (year 1 = 0.63; year 2 = 0.71; year 3 = 0.79) and, the ratios for *I*. *scapularis* based on 81 ticks were: 0.30:1 (overall), 0.29:1, 0.33:1, and 0.29:1 for years 1–3, respectively.

There were year-to-year differences in the number of ticks collected of all species and life-stages from all long-term sites, each representing predominantly woodland vegetation (Fig 4), predominantly grassland vegetation (Fig 5), and land cover with mixed vegetation (Fig 6). However, regardless of the land cover type, the phenology of all the tick species followed a unimodal density distribution during the 2014–2017 period, with peak densities of each species occurring during the spring and summer months (Figs 4–6).

Noticeable overlap in the questing activity of three post-emergent life-stages of *A*. *americanum* ticks was evident, with the nymphs emerging relatively earlier in the season followed by adults and larvae. In all years, and in all the sites, the peak activity of *A*. *americanum* nymphs occurred in May, adult activity peaked in June followed by larvae in August. Adult questing activity of *D*. *variabilis* increased in July, and the questing activity of adult *A*. *maculatum* occurred earliest in the year among all three ticks, in May, in all the sites. Male ticks of *D*. *variabilis* and *A*. *maculatum* that were collected from woodland vegetation quested in high numbers earlier than their female counterparts (Fig 4). Early emergence of male ticks was not observed among *A*. *americanum* ticks in any of the three vegetation types, and unlike the trend in woodland vegetation, the peak activity period for the adults of *D*. *variabilis* and *A*. *maculatum* in grassland (Fig 5) and mixed vegetations (Fig 6) occurred simultaneously. Interpretations on the phenology of *I*. *scapularis* ticks must be made with some caution due to the low number of ticks collected in this study. Nevertheless, the peak adult activity of the small number of these ticks occurred in October and November in woodland and mixed vegetation sites. There were no *I*. *scapularis* ticks found in the grassland in this study.

## 4. Discussion

This study has documented the diversity (Figs 2 and 3) as well as phenology (Figs 4–6) of different questing tick species in a Midwestern periurban environment—in areas where human and pet activities likely interface with the outdoor environment at a higher frequency. Increasing incidences of different TBDs in the midwestern US is an emerging concern, e.g., Beckham et al. [31]. The prevention and subsequent reduction of TBD incidences in a population relies on avoiding exposure to tick bites in the first place. Knowing which ticks are prevalent during which times of the year, as presented in this study, is an important first step towards achieving better prevention. Although differences in tick composition and activity periods may be expected in other regions of the Midwestern US, which is geographically quite expansive, we expect these findings are likely to be similar for most areas of eastern Kansas, western Missouri and northern Oklahoma and northern Arkansas due to the similarities in climate and land cover properties that these areas share. This study also provides a baseline information on tick diversity and seasonality for this region, which is often required but seldom available, for quantifying changes to tick ecology due to pressures exerted by non-stationary drivers such as climate change [32, 33].

All four tick species reported here are well known to researchers and have been recognized as medically- and/or veterinary significant for several years, if not decades. The prevalence and spatio-temporal distribution of TBDs in the study region, however, are only beginning to be quantified in the recent years [1–6]. An earlier survey by Raghavan et al. [34] in the same vicinity found different levels of tick-borne pathogen point-prevalence among ticks associated with different land cover types. However, the types of ticks prevalent in the area and their activity periods was not fully known. Questing tick species diversity noted in this study is consistent with other studies conducted over the years in this broad region [13, 19, 20, 35–37] although, the number of *A*. *maculatum* adults collected in the present study is higher than previously reported from areas closer to the study site [23, 37].

The questing activity of the three predominant tick species peaked once during the spring and summer months of each of the study year (Figs 4–6), and a distinct unimodal phenology for all life-stages and tick species is clearly evident. This finding is similar to Small et al. [13], wherein a unimodal distribution for *A*. *americanum*, *D*. *variabilis* and *I*. *scapularis* were found. However, their study was conducted over a single 12-month period and ticks were largely collected using CO_2_ traps and therefore any comparisons during the interannual periods cannot be made. The unimodal distribution found in the present study is also consistent with other longitudinal observations of tick activity in the broad region by other researchers [19, 20, 35, 36]. In two northeastern Missouri locations, approximately 250 miles northwest of the present study sites, Mangan et al. [27] continuously monitored the activity of post-emergent *A*. *americanum* life stages between 2007–2013. This study included *A*. *americanum* phenology data collected from the same two plots [38], previously reported by Bouzek et al. [25] and Kaizer et al. [26]. Adults and nymphs of *A*. *americanum* in these studies emerged concurrently in March—April, similar to our observation in all three predominant vegetation types. However, a second small second peak in nymphal activity was noticed in their studies. The modality of the seasonal pattern observed for each of the tick species, and in the case of *A*. *americanum* ticks the life stages, was consistent every year in the present study regardless of the predominant land cover type.

Although we observed only one peak adult activity period, interpretations of the phenology of *I*. *scapularis* need to be made with abundant caution because of the small number of these ticks we collected in the present study. A previous study by Kollars et al. [36] identified a bimodal distribution of *I*. *scapularis*, one occurring in November and another in March, from southeastern Missouri, an area not too far from the present study sites. The presence and potential westward expansion of *I*. *scapularis* in the Midwest is a concern. Not all areas in the broad study region (Kansas, Missouri, Oklahoma and Arkansas) currently have established populations of this tick, but the spatial distribution and abundance of *I*. *scapularis* could be steadily increasing in Missouri [39]. For instance, based on a long term study, recently Hahn et al. [28] reported an increase in the abundance of *I*. *scapularis* ticks collected both on- and off-hosts during a period between 2006–2015 from two northeastern Missouri locations [38]. Relative less abundance of *I*. *scapularis* ticks in the present study region may have prevented us from observing a second peak activity. Nevertheless, public health management strategies that can effectively prevent densities of these tick species in peri-urban areas, such as controlled burning of tick habitats and acaricide applications will benefit from utilizing the starting and end periods of tick activity described in this study.

Peak activity period for the different life-stages of *A*. *americanum* ticks occurred within a five-month period between April and August; however, the emergence of nymphs in March and the slow decline of larval activity toward the end of the season in October, in all three land cover types, shows a large window of activity period during which time these ticks are actively seeking bloodmeal hosts, albeit at different density levels. The pattern noticed for adult *D*. *variabilis* ticks are very similar to that of *A*. *americanum*, with a narrow peak but a broad host-seeking period ranging from April till October. The adults of *A*. *maculatum* ticks were active between March till July, shortest of the three predominant tick species found in the long-term sites. The mostly nidicolous immature stages of the latter two tick species were not collected using the methods we employed for this study, likely due to the larva and nymphs of these species living relatively closer to the ground and utilizing different hosts and host-seeking adaptations. It must be noted that quantification of activity period based on the collection of questing ticks alone represents only a portion of the ticks that are active under a certain set of conditions.

More female ticks of all four species were collected from the study region throughout the study period from all landcover/land use types. Prior studies conducted in the region on questing and host-attached ticks do not explicitly report sex ratios, but female-biased ratio can be generally seen in them [19, 20, 36, 37]. The skew towards more females was the strongest for *A*. *americanum*, with roughly only less than half the ticks being males, followed by *D*. *variabilis* with only a third of the total ticks being males. The reason for this consistent bias is not known to the authors but it could be related to adaptation in females to produce more daughters as few sons can fertilize many females, and therefore avoid inbreeding. Skewed ratios could also be caused by differential exposure to pathogens and subsequent higher mortality of male ticks. Laboratory studies to detect any reproductive parasites (e.g., Wolbachia, Rickettsia) and the differences in their prevalence for these individual tick species will help understand if this is a driving factor for the female bias.

One of the limitations of this study was our utilizing a tick collection method that only captured ticks that quested on vegetation. This likely led to us not finding the larval and nymphal stages of *D*. *variabilis* and *A*. *maculatum* as the larvae, and to some extent also the nymphs of these ticks live closer to the ground near the base of vegetation often in dense clusters, which are less likely to be captured by flagging method [40]. Immature *A*. *maculatum* ticks are particularly difficult to collect using the flagging method we employed [41, 42]. Nymphs and larva of *I*. *scapularis* was difficult to find likely due to their relative low abundance in the study area. Our purpose was to understand the diversity and phenology of questing ticks that pose a higher risk to humans and companion animals; however, future studies aiming to document tick phenology and diversity in this area will benefit by using CO_2_ traps and collecting host-attached ticks to get a fuller picture.

Our earlier studies in the study region on the spatiotemporal patterns of human monocytic ehrlichiosis [3], Rocky Mountain spotted fever [4], and bovine anaplasmosis [5], as well as the determination of potential ecological niche (spatial distribution) for the two vector species that are implicated in the transmission of these diseases, *A*. *americanum* [6, 7] and *D*. *variabilis* [8], are consistent with the relative abundance and phenology recorded in this study for these ticks. The significance of temporal effect in our studies that quantified incidence levels for these diseases [3–5] peaked around late summer and early Fall seasons, coinciding with the higher activity periods of nymphal and adult stages of *A*. *americanum* and adults of *D*. *variabilis* earlier in the season during spring and early summer. The detection of *I*. *scapularis* in this study is consistent with our previous prediction [9, 10] that the leading edge of spatial distribution for this species covers Pittsburg, Kansas and likely extend westward. Although the methods we used in that study did not allow us to predict the abundance or density for this species.

In conclusion, the predominant tick species found in and around periurban areas of Pittsburg, Kansas are *A*. *americanum*, *D*. *variabilis*, and *A*. *maculatum*, which are active throughout the spring and summer months of every year at varied levels of activity. The black legged tick, *I*. *scapularis* is also present in this area, in low densities compared to the other three tick species. These ticks are a hazard to human and companion animal health due to the pathogens they often transmit. Use of tick repellents while outdoors, staying away from vegetation at the edges of trails, checking oneself and companion animals for any attached ticks on return from outdoor activities, and prompt visit with healthcare provider if ticks are found to be attached will help prevent TBDs caused by these ticks.

## Supporting information

S1 File Number of ticks collected at the four seasonal sites around Pittsburg, Kansas, United States of America. (XLSX)

S2 File Number of ticks collected at the three long-term sites around Pittsburg, Kansas, United States of America. (XLSX)
